# Ventilation and Perfusion at the Alveolar Level: Insights From Lung Intravital Microscopy

**DOI:** 10.3389/fphys.2020.00291

**Published:** 2020-04-03

**Authors:** Jasmin Matuszak, Arata Tabuchi, Wolfgang M. Kuebler

**Affiliations:** ^1^Institute of Physiology, Charité – Universitätsmedizin Berlin, Corporate Member of Freie Universität Berlin, Humboldt-Universität zu Berlin, and Berlin Institute of Health, Berlin, Germany; ^2^The Keenan Research Centre for Biomedical Science at St. Michael’s, Toronto, ON, Canada; ^3^Departments of Surgery and Physiology, University of Toronto, Toronto, ON, Canada

**Keywords:** intravital microscopy, alveolar dynamics, alveolar perfusion, pulmonary microcirculation, acute lung injury, lung video microscopy

## Abstract

Intravital microscopy (IVM) offers unique possibilities for the observation of biological processes and disease related mechanisms *in vivo*. Especially for anatomically complex and dynamic organs such as the lung and its main functional unit, the alveolus, IVM provides exclusive advantages in terms of spatial and temporal resolution. By the use of lung windows, which have advanced and improved over time, direct access to the lung surface is provided. In this review we will discuss two main topics, namely alveolar dynamics and perfusion from the perspective of IVM-based studies. Of special interest are unanswered questions regarding alveolar dynamics such as: What are physiologic alveolar dynamics? How do these dynamics change under pathologic conditions and how do those changes contribute to ventilator-induced lung injury? How can alveolar dynamics be targeted in a beneficial way? With respect to alveolar perfusion IVM has propelled our understanding of the pulmonary microcirculation and its perfusion, as well as pulmonary vasoreactivity, permeability and immunological aspects. Whereas the general mechanism behind these processes are understood, we still lack a proper understanding of the complex, multidimensional interplay between alveolar ventilation and microvascular perfusion, capillary recruitment, or vascular immune responses under physiologic and pathologic conditions. These are only part of the unanswered questions and problems, which we still have to overcome. IVM as the tool of choice might allow us to answer part of these questions within the next years or decades. As every method, IVM has advantages as well as limitations, which have to be taken into account for data analysis and interpretation, which will be addressed in this review.

## Introduction

**FIGURE 1:**
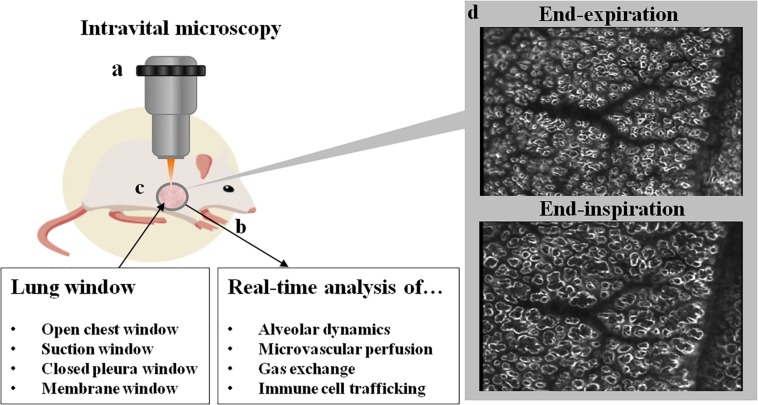
Schematic overview of a lung window and IVM. Intravital microscopy **(a)** is typically performed in mechanically ventilated animals (**b**; e.g., mouse, rat, swine, dog). Access to the lung is gained via excision of a surgical window **(c)** from the thoracic wall, with different types of windows currently in use. Imaging can be performed with several light microscopic applications ranging from widefield microscopy to multiphoton microscopy. In **(d)** two representative images of subpleural alveoli imaged by darkfield illumination at end-inspiration and end-expiration are shown. In the center of the image, a vascular tree can be seen with its terminal branches, surrounded by alveoli which distend during inspiration. On the right side of the image, the rim of the lung lobe can be seen. Ethical approval for the animal experiment was obtained from the Animal Care Committee of St. Michael’s Hospital Toronto. The figure was designed using resources from Freepik.com.

Intravital microscopy (IVM) is a two-dimensional imaging technique that allows for visualization of biological processes *in vivo.* It is highly versatile as it can be adjusted to various tissues and can be combined with high quality state-of-the-art microscopy enabling high resolution of tissue and single cells in the natural and complex environment of a multicellular organism (Figure 1). Some tissues in experimental animals are easily accessible, such as the vessels of the ear or can be easily exteriorized in mice for vascular IVM studies such as the mesentery or the cremaster muscle. For internal organs and/or chronic observation, the implantation of imaging windows is typically required, and widely used as abdominal, cranial and thoracic windows, as well as dorsal skin fold chambers. Depending on the desired penetration depth, the spatial resolution and the temporal resolution, different microscopic methods can be applied, ranging from widefield, laser scanning confocal, spinning disk, multi-photon microscopy or others. The use of fluorescent dyes and transgenic animals expressing fluorescent markers opens seemingly endless possibilities for the visualization of *in vivo* biological processes down to the subcellular level.

Albeit generations of excellent scientists have advanced our understanding of respiratory physiology to the current stage (Rahn et al., 1946; Suki and Bates, 2011), one of the most basic physiological principles, i.e., alveolar dynamics, is still surprisingly poorly understood. Our insight into the dynamic processes has been hampered by the limited accessibility of alveoli for dynamic studies at the microscale *in vivo* (Grune et al., 2019). Due to the lung’s complex structure, direct visualization by IVM is only possible for structures close to the lungs surface, such as subpleural alveoli. *In vitro*, as well as *ex vivo* or *in situ* models can only partially reflect the complex physiologic situation at the level of the alveolus and its surrounding capillary network in health and disease. Standard clinical and experimental imaging approaches including X-ray, computer tomography (CT), magnet resonance imaging (MRI), positron emission tomography (PET) and single photon emission computed tomography (SPECT) are able to image the whole lung and thus, provide important insight into disease state and general mechanisms, but are unsuitable to detect structures and processes at the microscale alveolar level. At the moment no non-invasive imaging technique is available that would enable the imaging of the alveolar or capillary microarchitecture *in vivo* at single-cell or subcellular resolution. The same applies to dynamic phenomena, such as alveolar dynamics, defined here as changes in alveolar size and morphology over the time course of the respiratory cycle in consideration of the underlying biophysical forces, or capillary microhemodynamics, i.e., microvascular flow characteristics and their determining factors in alveolar capillaries. Due to its anatomical complexity (Schneider et al., 2019) and dynamic behavior both at the level of ventilation and perfusion, direct imaging can provide unique insights into our understanding of lung physiology, perfusion and immunology, especially at the alveolar level. Yet, IVM of the lung simultaneously poses a series of unique problems, such as the restricted accessibility of the organ within an anatomical cavity at physiologically subatmospheric pressures. The three-dimensional structure of the alveolo-capillary unit comprises abundant air-liquid interfaces which prevent exploitation of e.g., multiphoton imaging for deep tissue penetration. The dynamic behavior of the lung tissue poses unique problems in terms of motion control. And finally its immunoprivileged status predilects the lung for immune reactions in response to surgical trauma or mechanical ventilation. Although IVM has been successfully used to study pulmonary microvascular responses as well as alveolar mechanics in real-time, the emerging understanding of alveolar physiology and pathophysiology are both still far from complete. Importantly, these limitations in our understanding are only in part attributable to a relatively small number of studies due to the complexity of such analyses. Adding to such limitations are controversial interpretations of the observations obtained from IVM. Such differences in interpretation relate in part to the above-mentioned methodological limitations of IVM, but also to the question how alveolar dynamics should be described, and what constitutes normal vs. pathological behavior, as will be discussed later in this review.

The key function of the pulmonary system is gas exchange, which is predominantly localized in the highly specialized respiratory zone, the alveoli. Interestingly, alveolarization and maturation of the microvasculature are the only developmental processes of the lung that continue after birth, up to 21 years (Schittny, 2017), emphasizing the importance and intricacy of the alveolar system. Alveoli are the dead-end of the respiratory tree and enable the uptake of oxygen and the removal of carbon dioxide at the alveolar membrane. To this end, the total alveolo-capillary membrane, comprised of the alveolar epithelial wall facing the gas side, the endothelial wall at the blood side, and a single fused basement membrane between the two, is extremely thin (at some places <100 nm thick). Almost 90% of the lung acts as a functional respiratory zone (parenchyma) with 300 million alveoli and an incredible surface area of 143 m^2^ (Gehr et al., 1978). The quality of gas exchange of the lung is directly dependent on the number of functional alveoli, the thickness of the alveolo-capillary membrane, alveolar vascularization and perfusion. In various lung diseases, this delicate alveolar design can be compromised leading to injured and dysfunctional alveoli and impaired gas exchange which in some cases will require supplemental oxygen or even mechanical ventilation. A prime example for such an acute scenario is the acute respiratory distress syndrome (ARDS), an acute failure of the respiratory system with an in-hospital death rate of approximately 30% (Petrucci and Iacovelli, 2007). ARDS is a multifactorial disease with various underlying causes, but is usually characterized by a rapid onset, an extensive inflammation of the lungs, and clinical characteristics such as failure of the alveolo-capillary barrier resulting in severely impaired gas exchange and abnormal low blood oxygenation (hypoxemia). In the absence of effective pharmacological treatments, mechanical ventilation is presently the mainstay of therapy to warrant oxygenation in ARDS. Yet, mechanical ventilation is a double-edged sword and has to be used with caution. On the one hand mechanical ventilation is essential for the patients’ survival, but on the other hand it can inflict permanent damage to the lung referred to as ventilator-induced lung injury (VILI) or ventilator-associated lung injury (VALI) (Amato et al., 1998), which negatively impacts both survival and post-survival life-quality. In already injured alveoli mechanical ventilation can impose further damage to the alveolar wall by physical and biological stresses, such as volutrauma, biotrauma or ergotrauma (which will be discussed in greater detail in the section: intravital microscopy for analysis of alveolar dynamics), although there is no consensus with respect to the main mechanism of injury.

Although – as will be discussed later – important limitations exist, IVM is currently the gold standard for visualization of alveoli *in vivo* and offers a broad potential to provide new insight into alveolar dynamics. In this review we will draw attention to the opportunities but also to the challenges and limitations of IVM in this context. We will discuss the use of IVM in different fields of lung research and provide a perspective for future areas of research. First, we will begin with a brief background on the development of IVM in lung research.

## Intravital Microscopy of the Lung – From Early Beginnings to Current State-of-the-Art

Although IVM of the lung will be considered by many as a very modern or recent technique, it was in fact applied as early as in 1661 by Marcello Malpighi. Malpighi observed the pulmonary microvasculature of the frog lung with a microscope and described for the first time alveoli and the surrounding alveolar capillaries (Young, 1929). More than 250 years later in 1925, Harry Hall visualized the pulmonary microcirculation in cats and rabbits by transilluminating the lung lobes outside of the chest (Hall and Chapman, 1925). Taking into account the importance of the natural physiologic environment of the lung, Hall’s approach to open up the chest and remove the lung for examination was successively replaced by a more elegant method, the thoracic window technique. The first attempt to create a thoracic window was accomplished by the removal of muscle from the rib cage with the parietal pleura being left intact (Wearn et al., 1934). The lung of the paralyzed animals was then transilluminated with a beam of light entering from a second window in the diaphragm. With this technique, Wearn et al. (1934) were able to describe the basis of capillary recruitment in the lung. The analysis of subpleural alveoli under closed thorax/chest condition was established in 1935 by Terry by implantation of a hollow metal cylinder centered by a cover glass (Terry, 1939). This revealed, however, a new problem of intravital imaging, namely the cyclic respiratory movements of the lung leading to motion artifacts. Wagner (1969) solved this problem in a canine model by a suction device which fixed the lung surface to a coverglass via negative pressure that was applied through a series of bore holes. This suction manifold was able to markedly reduce respiratory motion within the window of observation. Almost 50 years later, an improved lung window for stabilized imaging in mice was established by Looney et al. (2011) enabling live-imaging and in particular single cell tracking by two-photon imaging in an area of observation that is immobilized by a suction device. Vacuum suction stabilized lung windows are now widely used to analyze the pulmonary immune system (Looney et al., 2011). This includes neutrophil margination (Kuebler et al., 1994, 1997; Park et al., 2019) or immune cell – tumor cell interactions (Hanna et al., 2016), as well as infections of the lung (Sewald, 2018; Ueki et al., 2018).

Yet, although suction devices can effectively reduce or even eliminate motion artifacts in IVM, mechanical stabilization of the lung surface generates a non-physiological situation that prevents adequate assessment of alveolar dynamics and the effects of ventilation on microvascular perfusion. In addition, suction of the lung surface to a glass window for observation may potentially injure the sensible lung surface and stimulate inflammatory responses. To overcome these limitations, Tabuchi et al. (2008) developed a thoracic window without suction in which the lung is allowed to move freely within a closed chest cavity, thus mimicking physiological situations at the expense of image stabilization. In this approach, a thoracic window of 7 mm in diameter is generated by removal of chest muscles and ribs, and a transparent membrane is glued to the thoracic wall to re-create a closed-chest situation. With the help of a transdiaphragmal catheter the pleural air is removed and negative pleural pressure re-established, lifting the lung surface to the membrane-sealed lung window for microscopic observation. Although movements from respiratory and cardiac cycles persist in this model, identical areas will return into the microscopic focus plane during the inspiratory or expiratory plateau phase, allowing for ventilation-triggered imaging acquisition during these periods. By use of this approach Tabuchi and colleagues studied blood oxygenation (Tabuchi et al., 2013) and alveolar dynamics in healthy and diseased lungs (Mertens et al., 2009; Nickles et al., 2014; Tabuchi et al., 2016).

Similar to the “pleural window” by Wearn et al. (1934), Mazzuca et al. (2014) created an “intact pleural window” (size 0.5 cm^2^) by removing tissue down to the transparent parietal pleura allowing for direct visualization under closed-chest conditions without the implantation of a “artificial” lung window. This technique combines the advantages of the “physiological” free-moving lung in the Tabuchi model with a reduced surgical trauma at the expense of a smaller area of observation (i.e., one intercostal space). Other researchers adjusted their lung window by placing it in a small chamber at 37°C to exclude temperature-related adverse effects on the tissue directly located under the window surface (Kreisel et al., 2010).

All of the abovementioned lung windows are typically used for short-time imaging that does not exceed several hours. Only a few lung windows have been reported that may allow for long-term observations in dogs, rabbits (De Alva and Rainer, 1963), rats (Fingar et al., 1994) or mice (Kimura et al., 2010), albeit without stabilization and at low spatial resolution. Recently, however, Entenberg and colleagues developed an impressive model of a permanently implanted lung window in mice, which is minimally invasive, allowing the mouse to awake from the surgical intervention and breathe independently. For this approach the authors used an inert and corrosion-resistant steel window for high-resolution imaging of the lung (WHRIL) that allows for long term observation of the different stages of lung metastasis (Entenberg et al., 2018). According to this initial report, the window offers similar high-quality images as classic suction-fixed lung windows, with the possibility to visualize the same lung area repeatedly over several weeks by use of an established technique for microcartography (Entenberg et al., 2018). If this approach should be replicable in other labs and allow for longitudinal observations, it would open multiple new opportunities to monitor the progression of lung diseases. This would include changes in alveolar dynamics, microhemodynamics, microvascular tone regulation, and immune cell trafficking in animal models of different lung diseases including but not limited to ARDS/acute lung injury, lung fibrosis, COPD, pulmonary hypertension, and other parenchymal and vascular lung diseases.

Beside their “classic” use for intravital imaging, lung windows have also been effectively used to measure alveolar pressures in a closed-chest model in dogs (Bates et al., 1989), illustrating the versatility of the lung window approach. To this end, a small incision was made into the parietal pleura, and visceral and parietal pleura were glued together in this area. Next, a small plastic capsule was glued to the surface of the lung with a piezoresistive pressure transducer puncturing the visceral pleura for alveolar pressure measurement.

Despite the successful application of lung windows over several decades, some key limitations have to be taken into consideration for the study of alveolar dynamics. These restrictions relate in particular to four points, which will be discussed in greater detail in the chapter “Limitations of IVM”: First, IVM limitation to two-dimensional imaging, preventing three-dimensional analysis of alveolar dynamics. Second, IVM is presently restricted to structures at or close to the organ surface such as subpleural alveoli, while deeper lung compartments cannot be accessed. Third, IVM can only image and analyze a small percentage of the total number of alveoli. Fourth, the specific location at which IVM is performed may impact the findings, e.g., due to its position within the hydrostatic gradient of the lung (pulmonary bases vs. apex).

## Intravital Microscopy for Analysis of Alveolar Dynamics

Breathing is a complex process, in which air flows into the lung via the airways along a pressure gradient created by the contraction of the diaphragm and the external intercostal muscles. When external intercostal muscles and the diaphragm relax, lung elastic fibers and alveolar surface tension will cause a decrease in lung volume and thus, exhalation of carbon dioxide rich gas. Although the anatomical and physiological changes during the respiratory cycle at the level of the whole lung are well understood, there is presently no consensus on the corresponding tissue dynamics at the alveolar levels. Rather, different and in part mutually exclusive concepts and theories have been proposed how alveoli may behave during the physiological respiratory cycle, and in particular in diseased lungs, which will be described in more detail in the following paragraphs.

### Alveolar Dynamics in Healthy Lungs

In 1929 the physiologist Charles Clifford Macklin was one of the first to study the mechanics of the distal airspaces during the respiratory cycle. He assumed that the main volume change in the respiratory area during breathing occurs in the alveolar ducts and not the alveoli itself. This concept was based on the notion that alveoli experience almost no stretch, except for the border area between alveolar duct and alveoli. Rather, ventilation would be controlled by a continuous muscle system running form the larynx to the alveoli (Macklin, 1929). Approximately 40 years later this theory was questioned by experiments in feline (Storey and Staub, 1962) and guinea pig lungs (Forrest, 1970) where a rapid-freeze method was applied to analyze lungs at different volumes of inflation. Morphometric methods were applied to quantify the impact of lung volume change on alveolar and alveolar duct dimensions. These studies revealed an increase in alveolar volume (2×) and surface area (70%) when lung volume increased from functional residual capacity to 75–80% of total lung capacity (Storey and Staub, 1962) suggesting isotropic “balloon-like” distension of the alveoli. Subsequent studies by Gil and Weibel using electron microscopy of fixed rabbit lungs similarly suggested a change of alveolar volume as a function of total lung volume and explained it with an unfolding of alveolar septal pleats and folds and uncrumpling of the alveolar surface (Gil and Weibel, 1972; Gil et al., 1979). Notably, the notion of septal unfolding and uncrumpling – albeit providing an intuitive concept for the extraordinary capacity of the lung to accommodate considerable volume changes within the normal breathing range with relatively minor pressure increase – has at large been neglected over the past decade in the discussion of alveolar dynamics. While generations of scientists have studied the effects of mechanical stretch on alveolar epithelial cells using various commercially available or custom-designed stretch devices it is important to note that alveolar epithelial stretch – at least within the confines of normal tidal volumes – may be minimal if alveolar distension and contraction are primarily the result of surface folding/crumpling and unfolding/uncrumpling. Beside unfolding of alveolar septa and isotropic “balloon-like” changes of alveolar size, two other mechanism have been proposed as theoretically possible causes for the change of lung volume during inflation, namely a sequential recruitment of previously collapsed alveolar units or simultaneous changes in alveolar shape and size (i.e., non-isotropic distension) (Gil et al., 1979).

Importantly, the above reported findings were unanimously derived from morphological examinations and stereological analyses of excised and fixed lungs, prevented the analysis of the effects of different inflation levels within the same lung. More importantly, *post mortem* findings can only reflect static conditions yet not the dynamic behavior of alveoli over the respiratory cycle. Just as static and dynamic lung compliance diverge considerably; extrapolations from static end-point measurements of alveolar mechanics may lead to potentially erroneous interpretations regarding the dynamic behavior of alveoli in the intact lung. Important insights on such dynamic relationships was initially derived from theoretical models, notably the landmark paper by Jere Mead, who demonstrated the role lung distension and its relevance for the uniform expansion of air spaces (Mead et al., 1970). Other important topics addressed by theoretical models were stress-strain relationships (Fung, 1975), the dynamics of single vs. multiple alveoli (Schirrmann et al., 2010), as well as more complex alveolar regions (Tsuda et al., 2008; Sznitman et al., 2009).

Moreci and Norman (1973) performed the first *in vivo* observations of alveolar dynamics in a rat open chest preparation using incident light photomicrography. In this study, the authors observed a marked change in the size of the alveolar duct, whereas the alveolar volume only showed minor changes over the respiratory cycle. These findings were confirmed in a subsequent study from the same group (Daly et al., 1975) and would suggest the alveolar duct rather than the individual alveolus as the main site for airspace volume change. The group of Gary Nieman and colleagues, however, who performed an extensive series of IVM studies on alveolar dynamics in healthy and diseased lungs came to very different conclusions. At the end of the 1990s they proposed the concept of alveolar recruitment and derecruitment (R/D) in healthy dog lungs as the main mechanism for lung volume increase (Carney et al., 1999). Specifically, they proposed the recruitment (opening) of new (previously collapsed) alveoli when lung volume was increased from functional residual capacity (FRC) to 80% of total lung capacity (TLC). By IVM of dog lungs in an open chest approach via a glass coverslip that was carefully lowered onto the lung surface, the authors observed a small extent of isotropic alveolar distention during lung inflation, yet only up to 20% of TLC, with no detectable change in alveolar volume with further increases in lung volume. From these data and the observation that the number of alveoli per area of observation increased with higher lung volume, the authors concluded that changes in alveolar number due to recruitment rather than isotropic distension of alveoli (or alveolar ducts) must constitute the primary mechanism of physiological lung volume changes (Carney et al., 1999). In support of this notion, Hajari et al. (2012) calculated a linear increase in the number of alveoli with increasing total gas volume based on measurements by hyperpolarized ^3^He diffusion and MRI in healthy human subjects.

The concept of R/D as primary mechanism of lung volume change during the physiological respiratory cycle contrasts, however, with previous findings of alveolar distension and contraction as the predominant mechanism of volume change in healthy lungs (Storey and Staub, 1962; Forrest, 1970). Analogous to these latter reports, IVM studies of the mechanically unimpaired lung by use of the thoracic window technique developed by Tabuchi et al. (2008) identified alveolar clusters to expand gradually with increasing inspiratory pressure in ventilated mice. The resulting pressure-volume relationship for the individual alveolus followed a sigmoid curve (Mertens et al., 2009) that is largely reminiscent of the classic pressure-volume curve for the entire lung (Rahn et al., 1946). In contrast, the total number of alveoli per area of interest remained unchanged, indicating alveolar distension and contraction rather than R/D as the main mechanism of lung volume change in this study (Mertens et al., 2009). As IVM is always hampered by the extrapolation of two-dimensional observations to the three-dimensional stage, the latter study was further validated by parallel optical coherence tomography (OCT) which confirmed alveolar distension and contraction at the three-dimensional level (Gaertner et al., 2012).

While IVM in intact, live animals – ideally under minimally invasive, closed chest conditions or even under spontaneous ventilation – is the gold standard for the visualization of alveolar dynamics, a common alternative – albeit less physiological – model used to analyze alveolar dynamics that allows for optimal control of ventilation and perfusion are excised, mechanically ventilated and constantly perfused lungs. By this approach, Namati et al. (2008) observed alveolar distension during lung inflation, yet at the end of inspiration the number of alveoli increased while alveolar volume decreased. The authors interpreted this finding as evidence for R/D and developed the concept of secondary daughter alveoli that get recruited by the primary (mother) alveoli via the pores of Kohn, i.e., hole-like interfaces between neighboring alveoli, rather than the conducting airways (Namati et al., 2008). In subsequent studies the same group switched from isolated lungs to *in vivo* optical frequency domain imaging (OFDI) in swine. Remarkably, in these studies alveolar dynamics were best fitted by uniform alveolar distension, although a heterogeneous component of airspace distension was observed as well (Namati et al., 2013), yet no R/D. These two studies from the same group highlight exemplarily the impact of the chosen experimental model on the results generated. Accordingly, one crucial factor for discordant results in the field of alveolar dynamics might be based on the use of different experimental models and methods, which are further complicated by species differences.

Although in the past 50 years several different groups tried to shed light on alveolar dynamics with different models and methods, no overall consent has been developed and as such, the topic remains controversial. A recent editorial by Nieman (2012) discussing Hajari et al.’s (2012) analysis of alveolar dynamics by MRI-based ^3^He lung morphometry *in vivo* ensued a subsequent debate (Mitzner and Smaldone, 2012; Smaldone and Mitzner, 2012; Weibel et al., 2012) which summed up the different viewpoints and potential problems. As mentioned before Hajari et al. (2012) suggested that alveoli are regularly recruited and derecruited during inflation and expiration with no change of the inner surface area. Smaldone and Mitzner pointed out that these results, albeit showing parallels to their own work, critically depend on adjusting the parameters of a model to a single alveolar duct, thereby simplifying physiologic conditions (Smaldone and Mitzner, 2012). In the same discussion, Weibel highlighted that alveoli are often regarded as a unique structure itself, but that this is a misjudgment and the surrounding tissue and its interactions such as fiber tension and surface forces “make” alveoli. Schittny proposed that alveolar folding can be a proper explanation for recruitment in adult lungs and that R/D may be based on surfactant and surface tensions (Weibel et al., 2012). These different opinions highlight the current state of uncertainty with respect to alveolar dynamics, as – in the words of Smaldone and Mitzner – “what happens to lung acinar structure with lung inflation in a pathologic or even in a normal healthy lung remains unresolved” (Smaldone and Mitzner, 2012).

### Alveolar Dynamics in Diseased Lungs

The present lack of a consensus regarding normal alveolar dynamics constitutes one of the major obstacles for our understanding of pathological alveolar dynamics in various lung diseases. This is particularly relevant as an appropriate understanding of alveolar dynamics in the diseased lung is likely crucial for the development of optimized or personalized ventilation regimes and for the development of novel therapeutic strategies. The only current consensus on alveolar dynamics in diseased lungs is that they differ from normal alveolar dynamics and that this differential behavior will likely contribute to or aggravate VILI. The situation under pathologic conditions is further complicated by the expected heterogeneity of pathological alveolar dynamics. Alveolar mechanics under physiological conditions can be expected to be essentially uniform over time and even between species. In contrast, alveolar dynamics in injured lungs can be expected to be more heterogeneous at the regional or even local level (i.e., between neighboring alveoli), and may further differ depending on the etiology of ARDS (e.g., direct vs. indirect lung injury), its severity and time course, as well as the presence or absence of additional disease-related comorbidities.

The most popular concept for alveolar dynamics in the acutely injured lung postulates that alveoli become unstable, leading to collapse and reopening during spontaneous respiration and – in particular – mechanical ventilation. Several groups using a diverse set of methods have provided evidence in support of this concept. Taskar and colleagues ventilated rabbits with either healthy lungs (Taskar et al., 1995) or with lungs in which surfactant function had been impaired by inhalation of dioctyl sodium sulfosuccinate (DOSS) (Taskar et al., 1997) in an open chest setting. In both scenarios, ventilation with negative end-expiratory pressure (NEEP) caused R/D. In healthy lungs R/D as a consequence of NEEP ventilation caused a transient yet reversible decrease in arterial oxygenation and lung compliance, but no overt direct injury of the lung tissue on histological examination (Taskar et al., 1995). In contrast, in lungs with impaired surfactant function simulating an acutely injured lung, NEEP caused persistent severe tissue damage with impaired lung mechanics and gas exchange (Taskar et al., 1997). These effects were prevented by ventilation with positive end-expiratory airway pressure, suggesting that R/D may aggravate VILI in pre-injured but not necessarily in healthy lung. In line with these findings, Seah et al. (2011) showed in that a combination of high tidal volume and zero end-expiratory pressure (ZEEP) causes VILI due to R/D in a murine model, which was not the case when mice were ventilated with either high tidal volume or ZEEP. Based on these findings the authors postulated a “map” of “safe” and “unsafe” ventilation settings, highlighting that the “unsafe” area can dramatically widen in injured lungs. A time- and pressure-dependent effect on R/D was observed in saline-lavaged rat lungs following recruitment maneuvers (Albert et al., 2009). While rapid inflation occurred within 1–2 s, recruitment was notably delayed in that it was absent for the first seconds and then continued to progress over the entire measured time-frame of 40 s. This delayed recruitment response may be clinically relevant, as inspiratory time in the clinical setting is commonly in the range of 0.5–1.5 s, thus potentially preventing R/D in the clinical context.

In a different study, Neumann et al. (1998) tested three different injury models for their impact on alveolar dynamics as assessed by CT. After induction of lung injury by oleic acid, endotoxin, or lung lavage in six ventilated pigs, the authors measured lung density and observed a quick onset of R/D in all experimental models. The notion that R/D is a frequent form of impaired alveolar dynamics in injured lungs was further supported by a substantial number of IVM studies from the group of Gary Nieman. In one of their initial studies Nieman et al. (1981) demonstrated the importance of the surfactant stabilizing effect on alveoli in a canine model of surfactant deactivation by pulmonary lavage with Tween 20 or saline (as control), respectively. Surfactant deactivation by Tween lead to distinct changes in alveolar size, with total collapse at end-expiration, later defined as R/D. Within the 4 h of the experimental protocol, alveolar stability was restored. In subsequent studies with Tween-mediated surfactant deactivation in pigs the authors observed heterogeneous lung injury (Schiller et al., 2001) and progressive alveolar instability – notably defined as change in alveolar shape and size, see below – when tidal volume was increased from 6 to 12 and 15 mL/kg bw (Steinberg et al., 2002). A positive end-expiratory–pressure (PEEP) level of 10 cmH_2_O was able to stabilize alveoli, while lower PEEP levels resulted in R/D (Halter et al., 2003). In rats over-ventilated with an inspiratory peak pressure (IPP) of 45 cm H_2_O the authors observed alveolar instability or R/D after 45 min, which persisted for 90 min until the end of the study, yet contrary to what could be expected, gross damage to the alveolar epithelium was not observed. As such, the latter findings argue against a decisive role for R/D in the pathogenesis of VILI (Pavone et al., 2007). This notion is also supported by a series of preclinical and clinical studies suggesting that VILI is less pronounced in areas of cyclic R/D or atelectasis as compared to areas of high stretch and overventilation (Tsuchida et al., 2006; Bellani et al., 2011; Wakabayashi et al., 2014).

**FIGURE 2:**
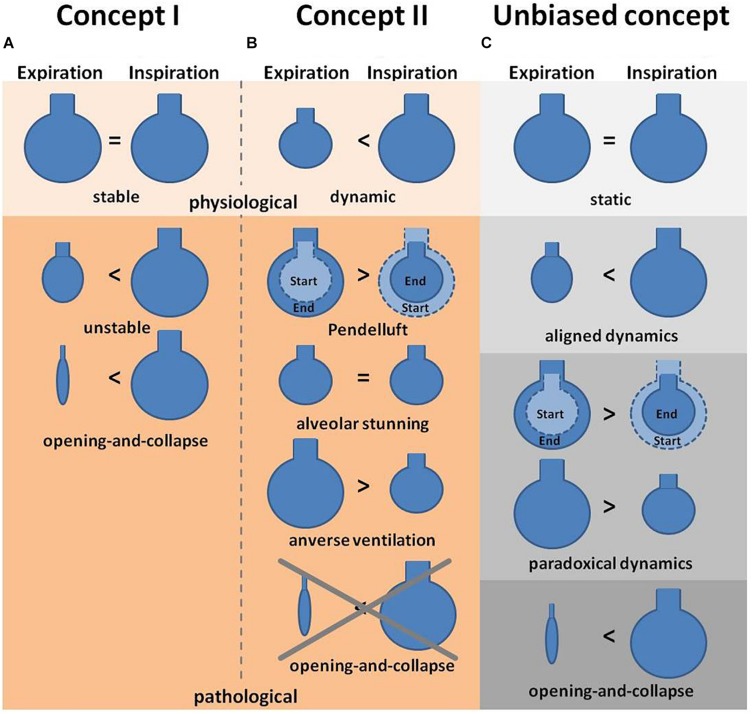
Concepts of alveolar dynamics. **(A)** Concept I describes alveoli under physiologic conditions as stable (i.e., with no change in volume) during expiration and inspiration, whereas pathological situations lead to alveolar instability (defined as size and/or volume change) or even opening-and-collapse of alveoli (Carney et al., 1999). **(B)** Concept II describes alveoli under physiologic conditions as alveoli that increase in volume during inspiration and contract during expiration, whereas pathological conditions are associated with an arrest of alveolar dynamics (alveolar stunning) or paradoxical motions, such as Pendelluft or inverse ventilation (Mertens et al., 2009; Tabuchi et al., 2016). **(C)** The proposed third concept is an unbiased terminology to describe alveolar dynamics. In this concept alveoli are classified as being either static (i.e., with no change in volume during ex- or inspiration), or showing aligned (volume increase during inspiration, decrease during expiration) or paradoxical (volume increase during expiration, decrease during inspiration) dynamics, or opening-and-collapse (i.e., complete volume loss during expiration). Notably this concept refrains from a classification into physiological vs. pathological dynamics.

In a model of surfactant depletion by Tween instillation Andrews et al. (2015) tested the possibility that R/D may be masked by a proper oxygenation rate, although alveolar dynamics are impaired. The group observed R/D, yet no decline in oxygenation, suggesting that the partial pressure of oxygen (PaO_2_) is not a parameter to identify impairment of alveolar dynamics, such as R/D. This important claim was subsequently validated by Allen et al. (2005) in a rat model of overinflation injury analyzed by pulmonary impedance measurements and *in vivo* imaging. They showed in their study that injurious mechanical ventilation leads to alveolar instability, but already before imbalances in the gas exchange are measurable. It is important to note, however, that the interpretation of these findings critically depends upon the underlying concept of what constitutes physiological vs. pathophysiological alveolar dynamics – and hence, upon a matter of considerable controversy (Figure 2). In the work by Andrews et al. (2015), R/D was defined as increase in alveolar size from expiration to inspiration – a behavior that many other studies would consider as physiological alveolar dynamics. Specifically, whereas some define distension of alveoli as a pathologic phenomenon, others interpret the distension from expiration to inspiration as the normal physiologic situation and no change in size as a sign for abnormalities. Conversely, as in the studies by Nieman cited above, a lack of change in alveolar size or shape is considered by some as physiological scenario and accordingly termed “alveolar stability”, whereas “alveolar instability” is used in these instances to describe any change in alveolar size or volume during the respiratory cycle. In contrast, other studies interpret alveoli with no volume change as pathological, and alveoli which distend and contract over the respiratory cycle [and would hence be considered as “instable” by Nieman et al. (1981), or as R/D according to Andrews et al. (2015)] as “normal” (Tabuchi et al., 2016). It goes without saying that these critical differences in terminology and interpretation have greatly contributed to the present lack of consensus on alveolar dynamics, and instead have added a substantial degree of confusion to the field. To limit the resulting ambiguity, we suggest here an unbiased terminology which categorizes alveolar dynamics into either “static” (i.e., no volume change), “aligned” (i.e., volume change in accordance with the parallel transpulmonary pressure changes), “paradoxical” (i.e., volume change that is not in accordance with the parallel transpulmonary pressure changes), and “opening-and-collapse” (R/D) (Figure 2). Notably, we refrain from a classification of these terms into physiological vs. pathological dynamics due to the ongoing controversy in this field. We hope that such a unifying terminology may ease comparability between future studies with the aim to ultimately allow for the development of a consensual concept.

In part, but not solely as a result of these ambiguities in terminology, the concept of R/D as postulated by the previously described studies has been opposed by a series of IVM studies that failed to observe any R/D under both physiological and pathological conditions (Mertens et al., 2009; Tabuchi et al., 2016). In a murine model of acid-induced lung injury a reduction of alveolar compliance was observed by dark field IVM that was especially pronounced in small alveolar clusters, thus causing a shift of alveolar ventilation to larger alveoli. Such a mechanism would amplify the heterogeneity of alveolar ventilation and thereby, promote alveolar overdistension (Mertens et al., 2009). Additionally, although no R/D was observed, several other “dyskinesias” such as pendelluft, alveolar stunning or reversed ventilation of alveoli were reported in different models of lung injury (Figure 2) including hydrochloric acid aspiration, Tween instillation for surfactant depletion and antibody mediated transfusion-related lung injury (Tabuchi et al., 2016). In all three models, the inflicted lung injury resulted in asynchronous alveolar ventilation, manifesting as alveolar pendelluft, as well as impaired gas exchange that was detected as local impairment of blood oxygenation. Notably, individual sighs of 10 s length were identified as an effective measure to reconstitute physiologic alveolar dynamics in some (acid-induced lung injury) but not all (Tween-injury) models of lung injury.

As such, considerable controversy exists regarding the characteristics and relevance of impaired alveolar mechanics in injured lungs in general, and the role of R/D in particular. Part of this controversy is attributable to different opinions on what constitutes “normal” alveolar dynamics and resulting differences in terminologies, definitions and interpretations. Methodological differences between analytical techniques (IVM vs. static) as well as specifics and limitations of the IVM technique itself (observation confined to subpleural alveoli, differences between hypostatic and apical lung zones, effects of alveolar edema) may contribute to seemingly opposing findings, while differences between species or models of acute lung injury are probably less likely to underlie these divergent findings.

## Intravital Microscopy for Analysis of Alveolar Perfusion, Gas Exchange, and Immune Cell Trafficking

Alveoli are the functional units of the lung with the overall task to warrant gas exchange, i.e., oxygen supply and carbon dioxide removal from the body. Besides alveolar ventilation, alveolar gas exchange is critically dependent on microvascular capillary network architecture and perfusion. Accordingly, capillary perfusion and its regulation have been a key interest for IVM studies in the past. In the late 1960s, Wagner (1969) developed a lung window with a suction device for dogs and applied it to analyze the pulmonary capillary transit time in local lung regions (Wagner et al., 1982). This development was a major advance, as before it had only been possible to measure the transit time for the entire lung which does not adequately reflect transit times in individual capillary beds. The measurement of pulmonary capillary transit time in dog lungs was realized by the injection of fluorescein isothiocyanate (FITC) labeled dextran into the corresponding lobe artery and subsequent microscopic observation of its passage through the pulmonary microvasculature. Notably, the mean capillary transit time was much longer than expected, namely 12.7 ± 3.2 (mean ± standard error) seconds in comparison to the 0.75 s for the whole lung capillary transit as measured by indirect methods. Wagner et al. (1982) concluded that this difference likely reflects the hydrostatic pressure gradient in the lung causing a similar vertical gradient of transit times.

Using a similar approach as Wagner, Fingar et al. (1994) implanted a transparent window chamber over the right lung lobe of Sprague Dawley rats to observe vessel permeability and edema formation in arterioles, capillaries and postcapillary venules. Pulmonary edema was induced by either oleic acid or compound 48/80 and measured by FITC-albumin leakage from the vessels into the alveolar space. Vascular leak was progressive over the observed time interval of 90 min and peaked after 30 min, with no measurable leak in control animals. Capillary leak from lung capillaries was similarly visualized by IVM as increase in alveolar septal thickness in a rabbit model of lung injury following complement activation by cobra venom factor (Kuhnle et al., 1999). The development of edema is a characteristic finding in ARDS that contributes critically to the propagation of VILI by diluting and inhibiting alveolar protective surfactant (Kobayashi et al., 1991), by reducing the fraction of aerated lung tissue that will consequently receive higher tidal volumes (“baby lung concept”) (Gattinoni and Pesenti, 2005), and by the development of transient liquid bridges which exert excessive shear stress to airway epithelial cells (Bilek et al., 2003; Huh et al., 2007).

In addition to the detection of edema formation, IVM has also been used extensively to characterize the processes of capillary recruitment and derecruitment (not to be confused with alveolar R/D discussed above). IVM studies in mechanically ventilated rabbits or dogs or isolated rat lungs have demonstrated the considerable potential for capillary recruitment in the pulmonary vasculature at high perfusion rates or pressures (Wagner, 1988; Hanson et al., 1989; Kuhnle et al., 1995; Baumgartner et al., 2003), respectively. Importantly, this topic has recently gained considerable translational attention since several clinical studies of pulmonary hemodynamics at rest and during exercise in healthy resting supine subjects suggest that most of the pulmonary microvasculature is already recruited at baseline. Specifically, these studies show that even a three- to fourfold increase in pulmonary flow results only in a small decrease in pulmonary vascular resistance (Argiento et al., 2012; Kovacs et al., 2012; Wolsk et al., 2017). As a consequence, the evidence for capillary recruitment in healthy, spontaneously breathing subjects remains inconsistent, and comparative studies using IVM (or similar techniques) under conditions of both mechanical ventilation and spontaneous breathing would be important to clarify this hallmark of classic lung physiology.

In addition to changes in capillary density, pulmonary vasoreactivity has been a subject of lung IVM studies, in particular focusing on the site and mechanism of hypoxic pulmonary vasoconstriction. Although IVM is limited to the visualization of blood vessels close to the lung surface, such as smaller arterioles and venules, it offers a versatile tool for the analysis of pulmonary vasoreactivity with unmatched temporal and spatial resolution in an intact anatomical and morphological context (McCormack et al., 2000). By use of the murine lung window technique Tabuchi and colleagues were able to monitor and quantify the vasoreactive response of pulmonary arterioles and venules as small as 20–30 μm in diameter to hypoxia (11% O_2_) or the thromboxane analog U-46619 in real-time (Tabuchi et al., 2008; Mertens et al., 2009). The typical vasoconstrictive response was predominantly located to arterioles with a diameter of 30–50 μm, less prominent in arterioles smaller than 30 μm and largely absent in venules. Hypoxic pulmonary vasoconstriction was similarly evident in pulmonary arterioles of the non-ventilated lung in rabbits undergoing one-lung ventilation (Groh et al., 1992).

**FIGURE 3:**
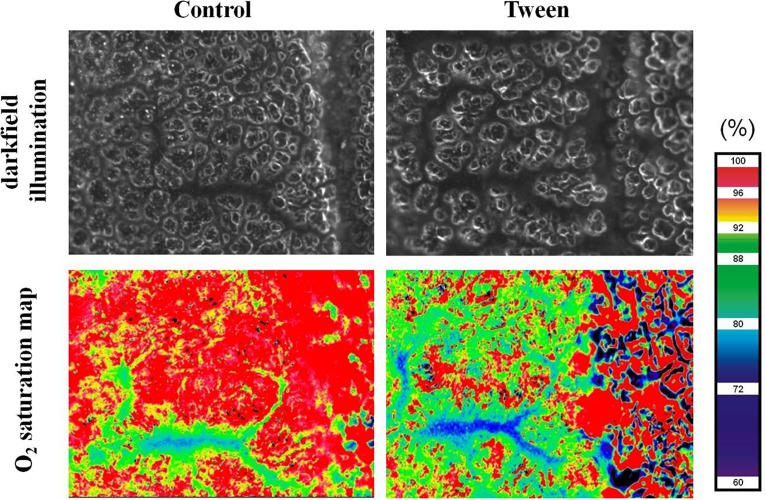
Oxygen saturation map. Representative images of a pulmonary arteriolar vessel tree in a ventilated mouse with the surrounding alveolar capillary network under control conditions and after treatment with tween imaged by darkfield microscopy (top) and multi-spectral oximetry resulting in the generation of a corresponding oxygen saturation map (bottom). Note impaired alveolar structure and oxygenation in the Tween model of acute lung injury. Ethical approval for the animal experiment was obtained from the Animal Care Committee of St. Michael’s Hospital Toronto.

In the early 2000’s, the group of Axel Pries developed a novel IVM technique to directly visualize microvascular oxygen saturation in individual vessel segments *in vivo* (Styp-Rekowska et al., 2007). To this end they utilized multispectral oximetry, i.e., the recording of entire absorption spectra over the wavelength range from 480–630 nm from the surface of a vascular network, subsequent pixel-based matching of the recorded spectra to the known absorption spectra of oxy- and deoxyhemoglobin, and vascular segmentation to ultimately generate oxygenation maps of intact microvascular networks. In combination with Tabuchi’s murine window technique, this approach allowed for the first direct visualization of the blood oxygenation process in the intact lung, an approach that surprisingly revealed that 50% of the total oxygen uptake at rest occurs in the precapillary arterioles before the blood enters the capillary network proper (Tabuchi et al., 2013). Combination of multispectral oximetry with IVM of alveolar dynamics and/or capillary perfusion as well as microhemodynamics yields a complex but versatile tool (Figure 3) that can provide unique insights into the process of alveolo-capillary gas exchange in the healthy and diseased lung (Tabuchi et al., 2016).

**FIGURE 4:**
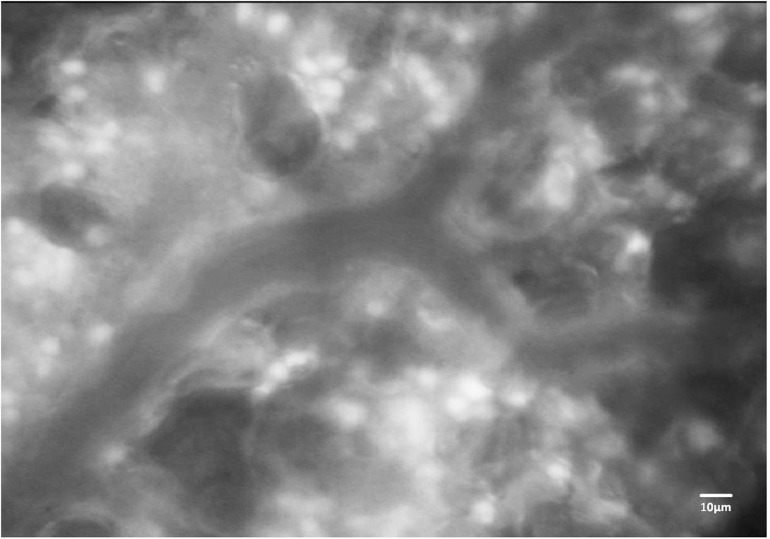
Fluorescence imaging of immune cells in the mouse lung. Representative image of a pulmonary microvessel after i.v. injection of rhodamine 6G. Note the accumulation of stained leukocytes in the alveolar networks outside of the larger pulmonary microvessel in the image center. Ethical approval for the animal experiment was obtained from the Animal Care Committee of St. Michael’s Hospital Toronto.

Leukocytes play key roles in inflammatory and immune responses of the lung. By use of IVM, the alveolar capillary bed has been identified as the main site of leukocyte retention during their passage through the lung (Lien et al., 1991; Kuebler et al., 1994), where they accumulate to form the so-called “marginated pool” (Kuebler and Goetz, 2002). This accumulation is attributable to the fact that the passing of leukocytes through the narrow capillary segments of the pulmonary microvasculature requires considerable neutrophil deformation, a process that has been termed neutrophil margination or sequestration (Doerschuk, 2001). Mechanical hindrance during the passage through the alveolar capillary network (Gebb et al., 1995; Kuebler et al., 1999) and the contribution of endothelial adhesion molecules and their interaction with leukocytes (Kuebler et al., 1997) constitute the predominant mechanisms that determine leukocyte sequestration and – upon appropriate chemotactic gradients – emigration into the alveolar space. Lien et al. (1991) suggested that leukocytes accumulate as a result of a misbalance between neutrophil delivery to the lung and divergent transit times along the capillaries. Kuebler and Kuhnle used IVM in a series of studies to identify the site of leukocyte sequestration (Kuebler et al., 1994), the effects of microvascular blood flow on leukocyte trafficking (Kuhnle et al., 1995), and the role of selectins in leukocyte margination (Kuebler et al., 1997). In addition to the dyes used for leukocyte labeling *in vivo* or *ex vivo*, these analyses were frequently combined with the use of other fluorescent dyes for concomitant analysis of edema formation by the assessment of vascular permeability, or for visualization and quantification of blood flow with plasma markers or labeled red blood cells. E.g., studies by Kuebler et al. (1994, 1997) and Kuhnle et al. (1995) in rabbits used FITC-labeled erythrocytes which were injected intravenously (i.v.) for quantification of blood flow velocity, red cell flux, and microhematocrit and i.v. administered rhodamine6G (Figure 4) as a marker for leukocytes with subsequent detailed analysis by IVM. Early on, the role of neutrophils within the pool of marginated and emigrating leukocytes has been of specific interest given their critical role in acute lung injury, pneumonia, and ARDS. Neutrophils not only play a critical role in the defense against pathogens, but also promote host tissue damage by the release of reactive oxygen species (ROS) (Simon et al., 1986; Smedly et al., 1986) thus aggravating edema formation and worsening outcome in ARDS patients (Steinberg et al., 1994). However, in early IVM studies the lack of specific dyes precluded a differentiation between different leukocyte subsets (Kuebler et al., 1994, 2000). In recent years, this limitation has been overcome by e.g., combinations of fluorescent dyes and antibodies to differentiate leukocyte subsets. To address the role of neutrophils in a model of sepsis-induced lung injury, Park et al. (2019) labeled erythrocytes *ex vivo* with DiD and reinfused them via a catheter. Concomitantly, vessels were visualized with FITC and Tetramethylrhodamine (TMR) conjugated dextran and neutrophils were specifically labeled *in vivo* with an anti-Ly6G^+^ monoclonal antibody tagged with a fluorophore. In this study, the authors confirmed the previously described retention of neutrophils within the pulmonary microvascular system (Doerschuk et al., 1987; Lien et al., 1987, 1991; Kuebler et al., 1994), but in addition could link it to an increase in dead space ventilation due to microvascular injury by ROS, ultimately causing impaired oxygenation and hypoxemia. Mark Looney and his group, which had revitalized the study of immune cells in the lung by their development of a stabilized murine lung window (Looney et al., 2011), performed a series of studies in which they addressed the kinetics of neutrophils in the lung. In a recent paper from 2018 they showed that the reduction of neutrophil extracellular trap (NET), a form of innate defense mechanism, by pharmacological or genetic tools resulted in less lung injury and improved outcome in a model of pneumonia induced by methicillin-resistant *staphylococcus aureus* (MRSA) infection. Conversely, in patients with ARDS high levels of NET formation were associated with increased mortality (Lefrancais et al., 2018). Studies by Yipp et al. (2017) identified a defense niche for neutrophils during infection with *Escherichia coli*, in that neutrophils can respond almost instantaneously via TLR4-MyD88 and abl tyrosine kinase signaling to gram-negative pathogens and endotoxins. These findings highlight the potential of IVM to unravel novel mechanisms of lung immunosurveillance and the roles of innate and adaptive immune cells in lung health and disease.

## Limitations of IVM

Intravital microscopy is a versatile tool that offers multiple possibilities to analyze biological processes in a realistic anatomical and physiological multicellular environment. IVM can be applied to a large variety of different organs, often in combination with other imaging modalities such as optical coherence tomography (Mertens et al., 2009; Gaertner et al., 2012). In case of internal organs or structures window techniques are commonly applied to provide visual access to the organ of interest such as the skeletal muscle. The lung, however, is an especially complex organ and difficult to image due to its inherent respiratory movements, the propagation of cardiac motion artifacts to the lung surface, as well as to the lung’s vulnerability to mechanical stress. Accordingly, preparation of lung windows without irritation of the lung surface and with maintenance of physiologic cardiovascular body functions (heart rate, arterial blood pressure, oxygenation, and cardiac output) throughout the experimental protocol with ongoing anesthesia and mechanical ventilation is challenging. Some lung windows are constructed to stabilize the lung surface for improved imaging with reduced motion artifacts. While this technique has proven highly efficient for tracking of immune cells, it will impair assessment of basic physiological phenomena, specifically alveolar dynamics and ventilation-dependent effects on lung perfusion due to the artificial removal of respiratory movements in the area of observation. In some studies IVM has been applied in open lung approaches by simply removing part of the thoracic wall and observation of the lung through this opening in the absence of a window. These approaches not only bear the risk of rapid drying of the area of observation and/or its exposure to artificial surfaces (such as cover slips) or environmental pathogens. It should also be considered that the mechanics of the thoracic wall and the interplay between intrapleural and transpulmonary pressures are fundamental factors of respiratory physiology. The disregard of these factors will have significant consequences on alveolar dynamics as well as microvascular perfusion and may contribute to the differential results obtained in studies of alveolar dynamics and the subsequent controversies.

In a pioneering study the group of Gary Nieman proposed that the volume change in healthy lungs is primarily based on the opening (recruitment) and collapse (derecruitment) of individual alveoli rather than the distension and contraction of alveoli (Carney et al., 1999). In this study which used an open chest technique the authors refrained from suction stabilization of the lung surface. Instead, a coverslip connected to the microscope objective was lowered onto the lung surface to generate a horizontal optical plane. While care was taken not to impact the underlying tissue, it seems difficult to conceive that this procedure would not lead to compression of the underlying alveolar structures. Other studies which used closed chest models in mice without suction applied to the lung surface, thus allowing for free movement of the lung, did not observe any R/D under physiologic conditions (Mertens et al., 2009), but instead described alveolar distension as the predominant mechanism of lung volume change.

While these discordant findings highlight how seemingly minor differences in experimental models and approaches may result in rather opposing results and interpretations, some general limitations that apply to all IVM models should not go unmentioned.

First, in many IVM models the lung surface is exposed to varying temperatures or room temperature, which will affect temperature-sensitive processes. Such processes are e.g., the activation of ion channels critical for the regulation of endothelial barrier function and neutrophil activation in ARDS (Goldenberg et al., 2015; Ahl et al., 2019). This can be avoided by the use of warmed lung window chambers (Kreisel et al., 2010) or simply by use of water immersion microscopy and continuous superfusion of the window with a pre-warmed solution (Kuhnle et al., 1993; Kuebler et al., 1994). Second, IVM of the lung is currently limited to the observation of subpleural alveoli, due to the restricted penetration depth of conventional fluorescence (approximately 30–50 μm), confocal (approximately 50–60 μm), and even two-photon microscopy (approximately 100 μm), therefore excluding deeper structures from analysis (Looney et al., 2011). Second, traction forces exerted by surrounding alveoli will differ between subpleural alveoli and deeper lung regions. Subpleural alveoli are on one side attached to the visceral pleura whereas in deeper lung regions alveoli are surrounded on all sides by other alveoli. Next, it should be considered that different regions of the lung differ in terms of ventilation and perfusion due to the vertical gravitational gradient. Consequentially, IVM observations, which are always restricted to a specific location, may differ considerably between lung base and apex. Both considerations (i.e., the problem of subpleural alveoli and the problem of different lung regions) relate to a more general limitation of IVM, namely the fact that the number of visualized (and analyzed) alveoli is commonly only a small fraction of the total alveoli in the lung and thus, not necessarily representative. Third, the limitation of IVM in terms of longitudinal studies should be considered. The majority of lung windows is too invasive to allow for long-term studies. The recently developed permanent lung window by Entenberg et al. (2018) is a notable exception, and may present a promising tool for future long-term observations. Fourth, albeit still the gold standard in terms of temporal and spatial resolution, IVM is a two-dimensional imaging method, giving only limited information about three-dimensional alveolar dynamics. While walls and outer diameter of the alveoli are commonly visible as bright reflections, it should be noted that these reflections can also result from capillaries or collagen in the pleura, making interpretations about the real alveolar dimension difficult. In healthy lungs air-filled alveoli appear as white ring structures in dark-field images; however, these rings are in fact the result of specific reflection processes, such as partial reflection and total internal reflection between neighboring alveoli. As a consequence, IVM dark-field images may result in an overestimation of actual alveolar size by a factor as big as ∼1.6 (Gaertner et al., 2015).

Optical coherence tomography, as a three-dimensional imaging modality albeit with a lesser temporal and spatial resolution as compared to IVM, avoids the above problem (Meissner et al., 2010) and further reveals another disadvantage of IVM, namely the inability to differentiate between collapsed or liquid filled alveoli (Gaertner et al., 2015). In ARDS the development of alveolar edema is a critical factor for impaired lung function and gas exchange, respectively, but its effect on alveolar dynamics is only partly understood. Edema fluid entering the alveolar space as a result of increased permeability of the alveolar wall accumulates in and ultimately floods entire alveoli. Alveolar flooding in turn impairs gas exchange and affects alveolar stability by diluting and inactivating surfactant, which functions as alveolar stabilizer by decreasing surface tension. In studies of alveolar micromechanics under pathologic conditions (such as instillation of the lung with liquid for mimicking edema, surfactant deactivation, or models of lung infection) cyclic disappearance and re-appearance of alveoli in IVM are commonly interpreted as R/D (Nieman et al., 1981; Schiller et al., 2001; Steinberg et al., 2002; Halter et al., 2003). In fact, however, this observation may similarly reflect fluid-filled alveoli becoming cyclically aerated and deaerated over the respiratory cycle. This was elegantly demonstrated by Gaertner et al. (2015) in a study directly comparing IVM and OCT imaging in fluid-filled lungs. Whereas in OCT the alveolar walls remained clearly visible (and showed open, yet fluid-filled alveoli), alveolar walls had seemingly disappeared in IVM images given the false impression of alveolar collapse.

## Conclusion

While IVM remains a key technology for the assessment of alveolar dynamics and perfusion, its inherent limitations necessitate validation by and combination with additional three- or even four-dimensional imaging techniques such as e.g., OCT. Only a combination of different imaging modalities, measurements of lung mechanics (e.g., by forced oscillations), and theoretical models can be expected to ultimately clarify the ongoing controversies in the field. Such a joint, interdisciplinary approach may not advance our understanding of respiratory physiology, but may promote the development of optimized and personalized ventilation modes based on a better understanding of alveolar dynamics and their impact on alveolar perfusion and gas exchange in healthy and diseased lungs.

## Ethics Statement

Ethical approval for conducted animal experiment was given by the Animal Care Committee of the St. Michaels Hospital Toronto.

## Author Contributions

WK, JM and AT contributed to the conception and design of the manuscript and wrote the sections of the manuscript. JM wrote the first draft of the manuscript. All authors contributed to the manuscript revision, and read and approved the submitted version.

## Conflict of Interest

The authors declare that the research was conducted in the absence of any commercial or financial relationships that could be construed as a potential conflict of interest.
